# Assessing the Molecular Targets and Mode of Action of Furanone C-30 on *Pseudomonas aeruginosa* Quorum Sensing

**DOI:** 10.3390/molecules26061620

**Published:** 2021-03-15

**Authors:** Victor Markus, Karina Golberg, Kerem Teralı, Nazmi Ozer, Esti Kramarsky-Winter, Robert S. Marks, Ariel Kushmaro

**Affiliations:** 1 Department of Medical Biochemistry, Faculty of Medicine, Near East University, Nicosia 99138, Cyprus; victor.markus@neu.edu.tr (V.M.); kerem.terali@neu.edu.tr (K.T.); 2 Avram and Stella Goldstein-Goren Department of Biotechnology Engineering, Ben-Gurion University of the Negev, Be’er Sheva 84105, Israel; karingo@post.bgu.ac.il (K.G.); esti.winter@gmail.com (E.K.-W.); rsmarks@bgu.ac.il (R.S.M.); 3 Department of Biochemistry, Faculty of Pharmacy, Girne American University, Kyrenia 99428, Cyprus; nazmiozer@gau.edu.tr; 4 The Ilse Katz Center for Meso and Nanoscale Science and Technology, Ben-Gurion University of the Negev, Be’er Sheva 84105, Israel

**Keywords:** halogenated acyl-furanone, furanone C-30, *Pseudomonas aeruginosa*, LasR protein, RhlR protein

## Abstract

Quorum sensing (QS), a sophisticated system of bacterial communication that depends on population density, is employed by many pathogenic bacteria to regulate virulence. In view of the current reality of antibiotic resistance, it is expected that interfering with QS can address bacterial pathogenicity without stimulating the incidence of resistance. Thus, harnessing QS inhibitors has been considered a promising approach to overriding bacterial infections and combating antibiotic resistance that has become a major threat to public healthcare around the globe. *Pseudomonas aeruginosa* is one of the most frequent multidrug-resistant bacteria that utilize QS to control virulence. Many natural compounds, including furanones, have demonstrated strong inhibitory effects on several pathogens via blocking or attenuating QS. While the natural furanones show no activity against *P. aeruginosa*, furanone C-30, a brominated derivative of natural furanone compounds, has been reported to be a potent inhibitor of the QS system of the notorious opportunistic pathogen. In the present study, we assess the molecular targets and mode of action of furanone C-30 on *P. aeruginosa* QS system. Our results suggest that furanone C-30 binds to LasR at the ligand-binding site but fails to establish interactions with the residues crucial for the protein’s productive conformational changes and folding, thus rendering the protein dysfunctional. We also show that furanone C-30 inhibits RhlR, independent of LasR, suggesting a complex mechanism for the agent beyond what is known to date.

## 1. Introduction

The traditional treatment of bacterial disease based on compounds that destroy or inhibit bacterial growth, though still useful, is no longer effective, in the long term, due to the high incidence of antibiotic resistance which has cost the world so much, in terms of morbidity and mortality. Quorum sensing inhibitors are currently being considered as potential alternative therapeutic interventions to antibiotics because of their ability to override bacterial infections without stimulating the incidence of resistance. Halogenated acyl-furanones, a class of secondary metabolites produced by an Australian marine red macroalga *Delisea pulchra*, are promising agents in the fight against bacterial infections in several species [1,2,3,4,5,6,7]. Studies suggest that these furanones, which are structural analogs to *N*-acyl homoserine lactones (AHLs), interfere with the swarming of bacteria, such as *Serratia liquefaciens* and *Proteus mirabilis*, as well as the bioluminescence emission of *Vibrio harveyi* and *V. fischeri* without affecting their growth [8,9]. The reported displacement of a radiolabel-AHL from LuxR [10] and the decrease of the LuxR protein concentration in the cell [6] suggest that the target of the natural furanone compounds could be the LuxR-type proteins. Thus, it is generally believed that furanones inhibit AHL-dependent QS by competing with AHL for the LuxR-type receptor site [6,7,8,10,11]. However, some studies propose alternate modes of action from the proposed competitive one in the case of furanone compounds [12,13].

Natural furanone compounds have no inhibitory activity against the notorious opportunistic human pathogen *Pseudomonas aeruginosa* QS circuitry [14]. Interestingly, two synthetic derivatives of the natural furanones, furanone C-30 [15] and furanone C-56 [16], lacking the alkyl side chain, have shown significant inhibitory effect against *P. aeruginosa* QS machinery. Yet, the molecular targets for these compounds and the precise mechanism of their actions remain poorly understood.

*P. aeruginosa* is an opportunistic pathogen that primarily infects persons with compromised immune systems, such as patients with acquired immunodeficiency syndrome (AIDS) [17], cancer [18], cystic fibrosis [19], and those with indwelling medical devices or burns [20]. In addition to co-regulating virulence, the QS signaling molecules produced by *P. aeruginosa* have been shown to have the ability to induce inflammation [21] and initiate pro-apoptotic immunomodulatory effects [22]. *P. aeruginosa* utilizes QS, a sophisticated system of communication depending on cell population density, to regulate multiple factors that are necessary for virulence and alteration of the host immune system [23,24,25,26]. At least three different QS systems, mediated by different signal molecules commonly known as autoinducers, have been identified in *P. aeruginosa*. Two of the systems, LasR-LasI and RhlR-RhlI, are interrelated and use AHLs as signals [24,25,27]. The third QS system, Pqs, uses an alkyl quinolone (AQ) as signal molecule [25,26,28,29]. It is generally accepted that LasR sits at the top of the regulatory hierarchy in the *P. aeruginosa* QS system, modulating the expression of several QS genes, including *rhlR* that codes for RhlR [24,30]. At a high cell population density, the receptors are activated by interacting with autoinducers to form regulatory complexes that result in the transcription of QS genes [26,31]. Altogether, LasR and RhlR regulate more than 600 genes, including 31 estimated regulators [23], thus providing *P. aeruginosa* with a significant advantage in causing pathogenicity.

The proposition that LasR is the master regulator in the *P. aeruginosa* QS cascade came from the study of laboratory strains [19,24,25,26,30]. Most studies, therefore, target LasR to attenuate *P. aeruginosa* virulence. Furthermore, *lasR* inactivation in *P. aeruginosa* clinical isolates from infected patients with advanced cystic fibrosis has been extensively reported [32,33,34,35]. The traditional regulatory hierarchy is altered in some clinical *P. aeruginosa* isolates, where RhlR regulation is independent of LasR regulation [19,35]. Interestingly, changes in *lasR* among the isolates from patients with acute infection are rare [32]. The inactivation of *lasR* suggests a loss of function of the master QS regulator. However, the inactivation of *lasR* in some clinical *P. aeruginosa* isolates might not have a severe impact on the *P. aeruginosa* virulence [36,37,38]. There have been reports showing that *lasR* mutants can cause an infection [39]. Some LasR-dependent phenotypes and products normally linked to virulence, including biofilm formation and protease synthesis, have been shown to remain among several *lasR* mutant clinical isolates [39,40]. Importantly, *lasR* inactivation confers on the clinical *P. aeruginosa* isolates an advantage with regards to growth as well as increasing its β-lactamase activity-enhancing tolerance against antibiotics [33]. These alterations observed in *lasR* mutant *P. aeruginosa* strains could offer novel opportunities in the ongoing effort for enhanced treatment of the disease. The RhlR protein, therefore, could be an important QS target for therapeutic intervention in clinical *P. aeruginosa* isolates since the master regulatory LasR is rendered redundant under some conditions [38]. Having an anti-QS molecule that can antagonize RhlR independent of LasR could have significant implications in the treatment against the opportunistic *P. aeruginosa* pathogen. In the present study, we provide evidence for the inhibitory action of furanone C-30 on *P. aeruginosa* LasR and RhlR receptors.

## 2. Results

### 2.1. Induction of Bioluminescence

Different concentrations of *N*-(3-oxododecanoyl)-l-homoserine lactone (3-oxo-C12-HSL, final concentration from 0.1 nM to 1 µM) and *N*-butanoyl-l-homoserine lactone (C4-HSL, final concentration 0.001–100 µM) were tested for maximum bioluminescence emission in PAO-JP2 (pKD201-*lasI*) and PAO-JP2 (pKD-*rhlA*) respectively [41]. Binding of 3-oxo-C12-HSL and C4-HSL to LasR and RhlR receptors generate regulatory complexes (LasR:3-oxo-C12-HSL and RhlR:C4-HSL) that drive the transcription of the QS genes [6,24], including the *luxCDABE* operon coupled downstream of the *lasI* promoter [41]. The results indicate that all concentrations used caused significant bioluminescence emission in the bioreporters, except for the lowest concerncentrations (Figure S1). Accordingly, 100 nM 3-oxo-C12-HSL and 10 μM C4-HSL exhibited the concentrations with the statistically highest significance.

### 2.2. Inhibitory Actions of Furanone C-30 on RhlR and LasR

**Figure 1:**
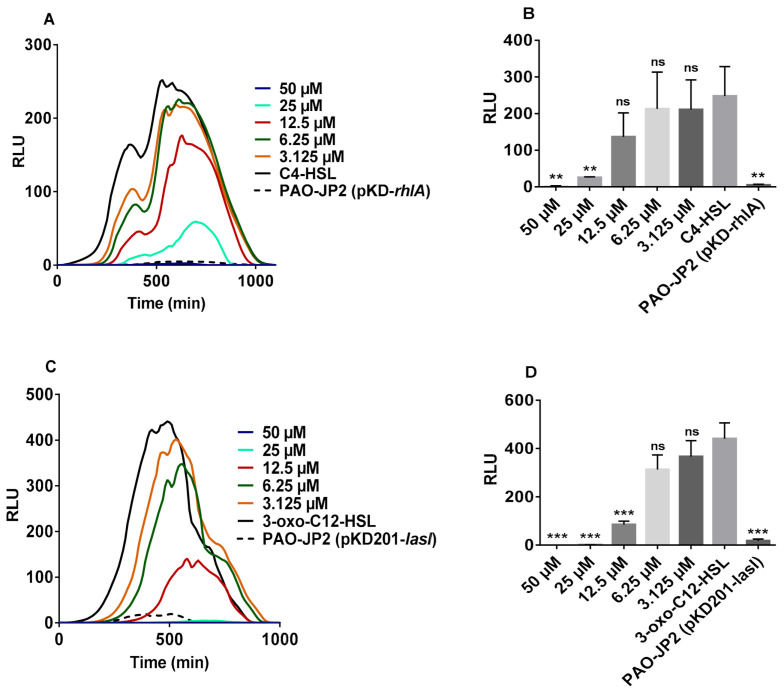
Inhibitory actions of furanone C-30 on RhlR and LasR. (**A**) Impact of furanone C-30 on RhlR. (**B**) RhlR inhibition relative to the control, correlating to the data in panel A. (**C**) Impact of furanone C-30 on LasR. (**D**) LasR inhibition relative to the control, corresponding to the data in panel C. The final concentration of 3-oxo-C12-HSL and C4-SHL were 100 nM and 10 µM, respectively. All concentrations presented are the final concentrations. Luminescence was expressed as relative luminescence units or RLU. ** *p* < 0.01, *** *p* < 0.001, and ns not significant. Values represent mean ± SD, *n* = 3 (three experimental readings).

Starting with PAO-JP2 (pKD-*rhlA*), a reporter strain containing RhlR-dependent QS, we assessed the inhibitory action of furanone C-30. In the absence of C4-HSL, only residual low light was emitted by the bioreporter. Introduction of C4-HSL induced the emission of intense light which was significantly decreased by furanone C-30 (Figure 1A,B). As expected, furanone C-30 significantly inhibited LasR (Figure 1C,D). The results suggest that furanone C-30 can inhibit RhlR independent of LasR. 

### 2.3. Swarming Motility Assay

**Figure 2:**
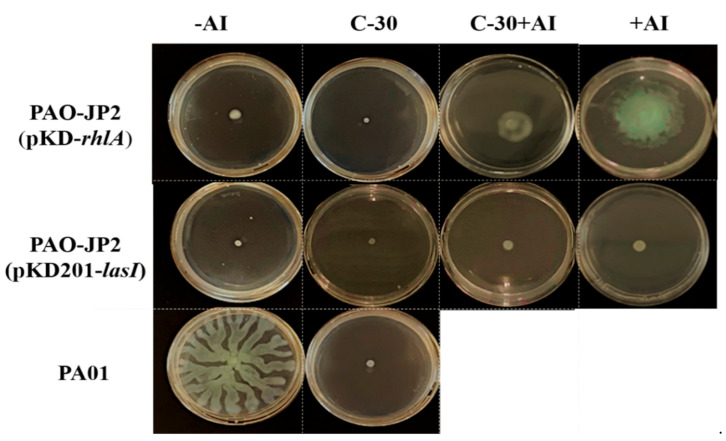
Images of *P. aeruginosa* motility in the presence and absence of autoinducers and furanone C-30. −AI indicates the absence of an autoinducer. +AI indicates the presence of an autoinducer. C-30 represents furanone C-30. The autoinducer for PAO-JP2 (pKD201-*lasI*) and PAO-JP2 (pKD-*rhlA*) were 3-oxo-C12-HSL and C4-HSL, respectively.

Since bacterial surface colonization is regulated via QS, we evaluated the inhibitory effects of furanone C-30 by conducting a swarming motility assay using the *P. aeruginosa* PA01 strain (wild-type), the *lasI-rhlI* double mutant strains (PAO-JP2 (pKD201-*lasI*) and PAO-JP2 (pKD-*rhlA*)). The results show that the *lasI-rhlI* double mutant strains were unable to swarm (Figure 2). Upon addition of the autoinducers, only the PAO-JP2 (pKD-*rhlA*) strain displayed swarming, while showing diminished swarming in the presence of furanone-C30. The wild-type strain exhibited swarming across the soft agar, but its motility was markedly inhibited in the presence of furanone C-30. These results suggest that *P. aeruginosa* surface colonization is controlled via RhlR regulation and that furanone C-30 effectively inhibits the C4-HSL-mediated QS system.

### 2.4. Effect of Furanone C-30 on LasR Solubility

**Figure 3:**
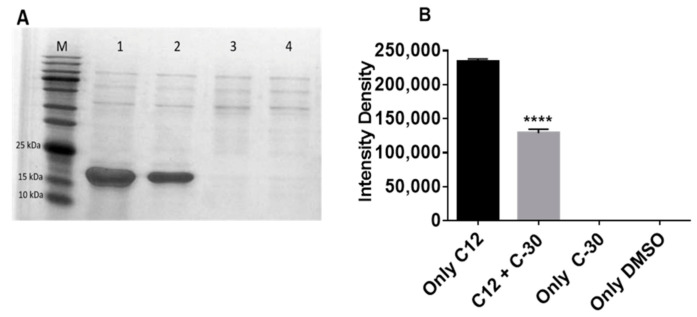
Effect of furanone C-30 on LasR solubility. (**A**) Image of LasR bands after SDS–PAGE, prepared using the ImageJ software [43]. C12 represents 3-oxo-C12-HSL and C-30 represents furanone C-30. M: Marker (10–180 kDa); Lane 1: C12 only; Lane 2: C12 + C-30; Lane 3: C-30 only; Lane 4: DMSO (1%, *v*/*v*) only. (**B**) LasR bands intensity density. The final concentration of 3-oxo-C12-HSL and furanone C-30 used were 100 nM and 50 µM, respectively. **** *p* < 0.0001.

Using *Escherichia coli* BL21 [42], LasR protein was purified in the presence of the 3-oxo-C12-HSL and/or furanone C-30. As shown in Figure 3A,B, no LasR protein was detected in the presence of 1% (*v*/*v*) dimethyl sulfoxide (DMSO) and furanone C-30. The presence of 7.5 μM 3-oxo-C12-HSL induced a significant expression of the LasR protein, but its concentration decreased significantly in the presence of furanone C-30. We could not exploit RhlR in our experimental setting, because of the difficulty in purifying the protein, possibly suggesting why there is still no crystal structure for this protein.

### 2.5. Prediction of Protein–Ligand Binding

**Figure 4:**
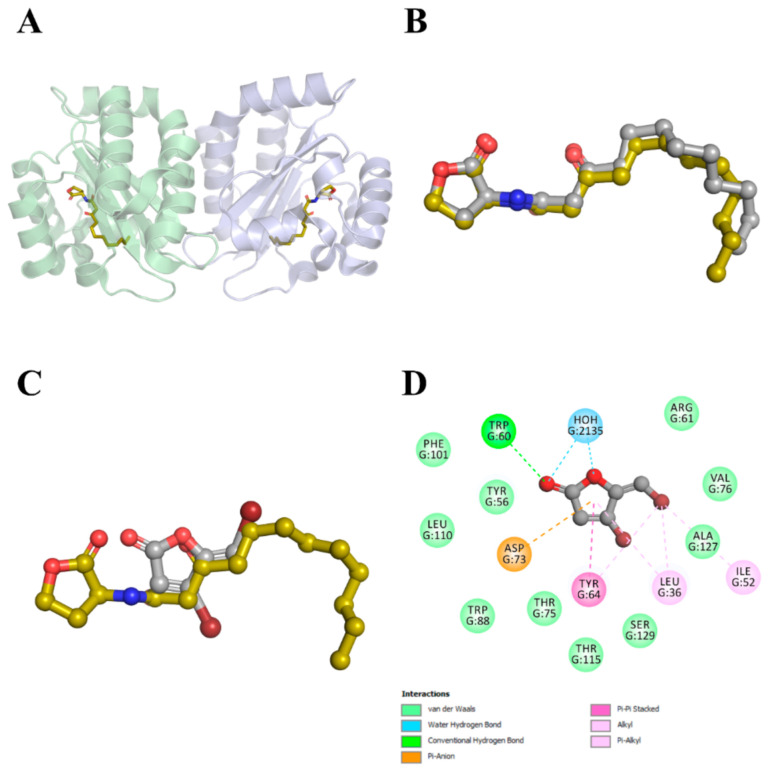
LasR–ligand docking and interaction profiling. (**A**) Ribbon representation of the biological assembly of the *P. aeruginosa* LasR ligand-binding domain, with two bound 3-oxo-C12-HSL molecules shown as gold sticks (PDB identifier: 2UV0). (**B**) Close-up view of the superposed structures of native (gold) and redocked (silver) 3-oxo-C12-HSL in the autoinducer-binding cavity of LasR (PDB identifier: 2UV0; chain identifier: G). (**C**) Close-up view of the superposed structures of native 3-oxo-C12-HSL (gold) and cross-docked (silver) furanone C-30 in the autoinducer-binding cavity of LasR (PDB identifier: 2UV0; chain identifier: G). (**D**) Favorable non-covalent interactions that are predicted to occur between furanone C-30 and LasR. The images (**A**–**C**) were rendered using the PyMOL Molecular Graphics System, v1.8 (Schrödinger LLC, Portland, OR, USA). The image (**D**) was rendered using Discovery Studio Visualizer, v16.1.0 (Dassault Systèmes BIOVIA Corp., San Diego, CA, USA).

The autoinducer-binding domain (residues 16–161) of *P. aeruginosa* LasR is a symmetrical dimer, with each monomer displaying an α-β-α fold that binds one molecule of 3-oxo-C12-HSL (Figure 4A). Further, 3-Oxo-C12-HSL has been reported to engage in hydrogen-bonding interactions with Tyr56, Trp60, and Ser129 through its carbonyl groups, as well as, with Asp73 and Thr75 through its amide group. In addition, a water molecule seems to bridge the Arg61 and the 3-oxo carbonyl group. The long acyl chain of the reference ligand is accommodated in a large hydrophobic pocket on the other side of the autoinducer-binding site [44]. The results of redocking calculations demonstrated that JAMDA was able to reproduce the native binding pose of 3-oxo-C12-HSL well, with a root-mean-square deviation (RMSD) of 0.917 Å and a JAMDA score of −3.15263 (Figure 4B). Cross-docking calculations revealed that furanone C-30 could be housed successfully in the active-site cavity of LasR, albeit with a comparatively lower JAMDA score (−1.18356). Upon a closer look, the physicochemical and geometric properties of the favorable interaction spots of furanone C-30 appeared to match those of 3-oxo-C12-HSL in the LasR-bound state (Figure 4C). Furanone C-30 was found to be stabilized mainly by hydrogen-bonding, electrostatic and hydrophobic interactions. Although it managed to establish favorable non-covalent interactions with Trp60 (hydrogen-bonding interaction at 2.55 Å), Asp73 (anion–π interaction at 3.46 Å) and several hydrophobic residues, it failed to interact with the other critical residues that are known to play an important role in promoting the proper folding and solubility of LasR (Figure 4D). 

**Figure 5:**
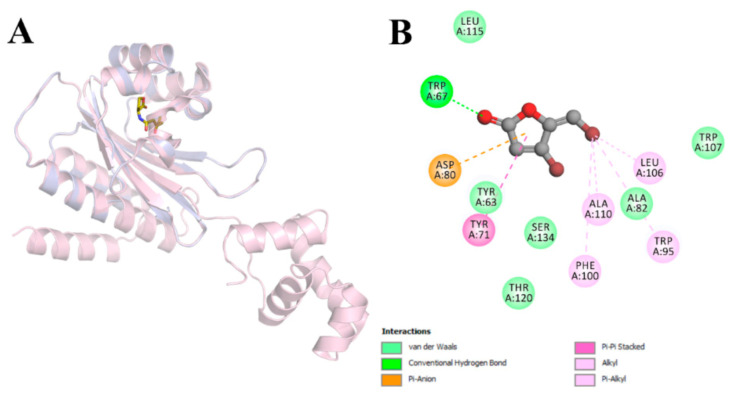
RhlR–ligand docking and interaction profiling. (**A**) Cartoon view of the superposed structures of the *E. coli* SdiA monomer (light pink; PDB identifier: 4Y15) and *P. aeruginosa* RhlR ligand-binding domain (light blue; homology model). The SdiA-bound exogenous ligand 3-oxo-C6-HSL is shown as gold sticks. This image was rendered by using the PyMOL Molecular Graphics System, v1.8 (Schrödinger LLC, Portland, OR, USA). (**B**) Favorable non-covalent interactions that are predicted to occur between furanone C-30 and RhlR. This figure was rendered by using Discovery Studio Visualizer, v16.1.0 (Dassault Systèmes BIOVIA Corp., San Diego, CA, USA).

Earlier studies showed that SdiA, which represents the LuxR-type receptor for AHL detection found in *E. coli* and *Salmonella*, is most closely related to RhlR, suggesting that *sdiA* was acquired by the horizontal gene transfer of *rhlR* from a pseudomonad [45,46]. Therefore, we homology-modeled the autoinducer-binding domain (residues 23–166) of *P. aeruginosa* RhlR based on the crystal structure of *E. coli* SdiA complexed with 3-oxo-C6-HSL (PDB identifier: 4Y15) [47]. The autoinducer-binding domain of *P. aeruginosa* RhlR shares a sequence identity of 34% (38% similarity) with that of *E. coli* SdiA, indicating to the correct fold of the resulting model (Figure 5A). Here in the active-site cavity of RhlR, furanone C-30 appeared to adopt a binding pose (JAMDA score: −1.12827) similar to that we observed in the case of LasR–furanone C-30 binding, as revealed by docking simulations (Figure 5B).

## 3. Discussion

Microarray analysis suggests that the gene clusters, *lasI*-*lasR*, and *rhlI*-*rhlR*, which encode central QS regulatory components in *P. aeruginosa*, were not markedly affected by furanone C-30 [15,48]. This indicates that the inhibitory effects of furanone C-30 on *P. aeruginosa* QS may not be at the gene level but likely at the post-transcriptional level. In the present study, we demonstrate that furanone C-30 exerts significant inhibitory activity against both LasR and RhlR. We also show that furanone C-30 inhibits RhlR independent of LasR, suggesting a complex mechanism for the agent beyond what has been previously known. Furanone C-30 (up to 50 µM) has been shown to have no observable restraint on bacterial growth [49]. The concentration range used in this study was 3.125–50 µM.

In *P. aeruginosa* infections, motility plays an important role in the surface colonization and the ensuing establishment of biofilms [50]. Rhamnolipid, a biosurfactant that induces swarming motility in *P. aeruginosa*, is excessively produced when the RhlR-dependent QS system is upregulated [51,52]. Because furanone C-30 showed a significant inhibitory impact on RhlR in our reporter assay, we hypothesized that similar impacts might also be seen in bacterial surface colonization. Indeed, we demonstrated that *P. aeruginosa* surface colonization is controlled via RhlR regulation and that furanone C-30 effectively inhibits the C4-HSL-mediated QS system. These results stressed the inhibitory impact of furanone C-30 on the RhlR-dependent QS and, specifically, its effects on the several phenotypes controlled by the RhlR receptor. Previous studies have considered the RhlR QS receptor as an emerging target for *P. aeruginosa* virulence control. Eibergen et al. demonstrated how some synthetic compounds selectively modulate RhlR instead of LasR and QscR [53]. Furthermore, functions commonly known to be dependent on LasR regulation such as the production of 3-oxo-C12-HSL and elastase B have been observed in *lasR* mutant *P. aeruginosa* [36,38,54]. Since the strains lacking both the *las* and *rhl* systems lost all functions, Dekimpe and Déziel [54] concluded that RhlR regulates the *las* regulon. These data, therefore, suggest that the influence of RhlR regulation may be beyond the generally known RhlR regulon.

To further assess the inhibitory action of furanone C-30 on AHL-mediated QS, we purified LasR in the presence of the 3-oxo-C12-HSL and/or furanone C-30. Our results show that furanone C-30 significantly decreased the concentration of the LasR protein. This indicates that furanone C-30 anti-QS activity may not completely wipe out infection, but it could render the pathogen prone to the effects of bactericides and clearance of the host immune defense system. In concert with our findings, some previous studies have shown that furanone C-30 may hold exciting prospects in combination therapy. This molecule has been demonstrated to increase the susceptibility of *P. aeruginosa* biofilm to antibiotics [15,55] and enhanced *P. aeruginosa* clearance from mouse lungs by the immune defense system [15].

In the presence of furanone C-30 only, there was no LasR protein in the cells’ supernatant, thus indicating that the protein was not soluble. This finding suggests that furanone C-30 is unlikely to engage in favorable/adequate interactions with the autoinducer-binding residues of LasR to retain the stability of the receptor. Some LasR competitive inhibitors (such as *meta*-bromothio lactone) have been shown to cause LasR to remain in solution [56,57]. Our findings appear to agree with the report of Bottomley et al. [44]. Using a powerful technique (NMR spectroscopy) that can efficiently detect protein–ligand interactions, Bottomley et al. [44] found no soluble LasR:furanone C-30 complex or evidence of furanone C-30 binding to the already soluble LasR:AHL complex. Several anti-QS molecules against Gram-negative bacteria have been reviewed [58]. Several of these molecules were from the marine environment. In the complex marine environment, competition may exist among the bacterial populations so that the dominant communities synthesize and secrete compounds that disrupt the QS signaling of others as part of the strategy and mechanism of survival [59,60]. There is evidence that even marine organisms, such as sponges, harbor molecules that inhibit QS. Indeed, compounds such as manoalide, manoalide monoacetate, and secomanoalide from the marine sponge *Luffariella variabilis* have been reported to significantly inhibit *P. aeruginosa* LasR and RhlR [61]. Patulin and penicillic acid produced by fungi were also found to possess significant inhibitory effects against *P. aeruginosa* AHL-mediated QS [62]. Both patulin and penicillic acid are structural analogs to furanone C-30. As demonstrated using furanone C-30, Carfi and co-workers found no soluble complex of LasR with patulin or penicillic acid [44]. Seeing that there are no alternative sites on the surface of LasR for allosteric interactions, the authors conclude that furanone C-30, patulin, and penicillic acid inhibit LasR by competing with AHL, thus interfering with the folding and solubility of the protein.

The binding of autoinducers to LuxR-type receptors has been suggested to create stability for the nascent proteins, followed by the dimerization of the protein, and then binding of the regulatory complex to the promoter DNA that drives the transcription of QS target genes [6,24]. Favorable interactions with certain residues lining the ligand-binding site can determine whether autoinducer analogs and other QS modulators favor agonism or antagonism. These residues include Tyr56, Trp60, and Ser129 [63], as well as Tyr64, Val76, Trp88, Leu125, and Ala127 [64]. In addition, O’Reilly et al. [65] indicated the crucial role of a flexible loop (conventionally called L3) that enhances specific conformational changes upon the binding of potent agonists in the ligand-binding pocket. This flexible loop (residues 40–51) is responsible for closing the ligand-binding site against bulk solvent via the packing of Tyr47 [66,67] enabling LasR to house ligand molecules with larger substituents (e.g., longer acyl chains). In our study, the decrease in LasR solubility by furanone C-30 in the presence of 3-oxo-C12-HSL implies that the interaction between LasR and 3-oxo-C12-HSL was impaired. We did not go further to evaluate LasR dimerization and DNA binding in the presence of furanone C-30 because our observation suggests that these processes are preceded by the binding of LasR to 3-oxo-C12-HSL. In tandem with the observations in previous studies, our findings suggest that furanone C-30 interacts with LasR to interfere with the conformational changes required per se for correct protein folding, thus rendering LasR dysfunctional and likely enlisting it for aggregation and proteolytic destruction. 

The results of docking calculations show that furanone C-30 is likely to occupy the active-site cavities of LasR and RhlR. Interestingly, our predicted LasR:furanone C-30 complex is different from the model proposed by Bottomley et al. [44]. Although the HSL ring of 3-oxo-C12-HSL is somewhat structurally analogous to the γ-crotonolactone (GCL) ring of furanone C-30, it is believed that even minor variations in the structure of a potent QS modulator can have distinctive and notable impacts on QS signaling in microbial communities [68]. For example, recent extensive structure-activity relationship studies of autoinducer analogs have demonstrated that the replacement of the HSL moiety (present in the native autoinducer) with a substituted aromatic system alters the agonistic activities of the molecules towards antagonistic activities [69,70,71]. Accordingly, it is tempting to assume that the conjugated (non-aromatic) π system and lipophilic bromine atoms of furanone C-30 confer the inhibitor with the pharmacophore features that are required by LasR (and RhlR) for QS-modulator reception. They, however, fail to establish the sufficient number of interactions with the surrounding active-site residues to generate a soluble complex with productive folding and hence full biological activity.

Overall, we demonstrate that furanone C-30 possesses the ability to inhibit both LasR and RhlR independently. To date, furanone C-30 was thought to inhibit *P. aeruginosa* QS only via the LasR receptor. The inability of furanone C-30 to establish interactions with the residues critical for generating a soluble complex makes it a potent anti-QS molecule. Our results also add to the relevant scientific literature indicating that RhlR holds promise as a potential target for treatment against clinical *lasR* mutant *P. aeruginosa* isolates, and it further emphasizes the therapeutic potential of the anti-QS agents in the ongoing fight against bacterial infections. 

## 4. Materials and Methods

### 4.1. Materials

Difco Luria-Bertani (LB) Broth, Miller (10 g L^−1^ tryptone; 5 g L^−1^ yeast extract; 10 g L^−1^ NaCl) and Difco LB agar, Miller (10 g L^−1^ tryptone; 5 g L^−1^ yeast extract; 10 g L^−1^ NaCl; 15 g L^−1^ agar) obtained from Becton (Dickinson & Company, France). Phenylmethanesulfonyl fluoride (PMSF), lysozyme, IPTG, Na_2_H_2_PO_4_, kanamycin A monosulfate, trimethoprim, 3-oxo-C12-HSL, C4-HSL, and furanone C-30 were purchased from Sigma-Aldrich (St. Louis, MO, USA). Glycerol, NaCl, 10–180 kDa molecular mass marker, acrylamide/bisacrylamide 37.5:1 (40%, *w*/*v*) were purchased from Bio-Lab (Hanapach Ashkelon, Israel). Instant Blue (a Coomassie-based staining preparation for protein gels) was procured from Expedeon (Cambridgeshire, United Kingdom). Sample (loading) buffer for SDS–PAGE was purchased from Carl Roth (Karlsruhe, Germany). DMSO was obtained from Fisher Scientific (Leics, United Kingdom). Imidazole was obtained from Alfa Aesar (Thermo Fisher Scientific, Kandel, Germany). 

### 4.2. Bacterial Strains and Plasmids

PAO-JP2 (pKD201-*lasI*), a *lasI-rhlI* double mutant of *Pseudomonas aeruginosa* PAO1 that harbors pKD201 vector with *lasI* promoter coupled upstream to the *luxCDABE* box. PAO-JP2 (pKD-*rhlA*), a *lasI-rhlI* double mutant of *Pseudomonas aeruginosa* PAO1 that harbors pKD vector with *rhlA* promoter coupled upstream to the *luxCDABE* operon. *Escherichia coli* BL21 strain transformed with a pETM-11 vector encoding for His_6_-tagged LasR construct (LasR-LBD) spanning residues Met-1 to Lys-173. All strain stocks were graciously provided by Prof. Meijler M. M., Ben-Gurion University of the Negev, Israel. 

### 4.3. Strain Cultivation

The PAO-JP2 (pKD201-*lasI*) and PAO-JP2 (pKD-*rhlA*) strains were cultured on LB-agar plates supplemented with 300 μg mL^−1^ trimethoprim for 24 h at 37 °C in the incubator (Binder, Camarillo, CA, USA). A starter culture was prepared by introducing a single colony into 10 mL LB broth containing 300 μg mL^−1^ trimethoprim (with the cap of the tube half-open stabilized with autoclave tape) and grown overnight at 37 °C with agitation (140 rpm) on a rotary thermo-shaker (Gerhardt, Germany). Also, the *E. coli* BL21-pETM-11 strain was cultured on LB-agar plates containing 50 μg mL^−1^ kanamycin for 24 h at 37 °C in the incubator (Binder, Camarillo, CA, USA). A starter culture was prepared by introducing a single colony from the LB-agar plate into 5 mL LB broth containing 50 μg mL^−1^ kanamycin and grown for approximately 17 h (with the cap of the tube half-open and stabilized with autoclave tape) at 37 °C with agitation (140 rpm) on a rotary thermo-shaker (Gerhardt, Germany). A 50 mL fresh portion of LB medium containing 50 μg mL^−1^ kanamycin was inoculated with 500 μL of the starter culture and re-grown at 37 °C for about 2–2.5 h with agitation (150 rpm) till OD_600_ = 0.4 measured using Ultrospec 2100 pro spectrophotometer (Amersham, Berks, England). The respective LB-agar plates containing PAO-JP2 (pKD201-*lasI*), PAO-JP2 (pKD-*rhlA*), and *E. coli* BL21-pETM-11 strains were stored at 4 °C for future use, not more than one month. 

### 4.4. Induction of Bioluminescence

Different concentrations of 3-oxo-C12-HSL and C4-HSL were tested for the induction of PAO-JP2 (pKD201-*lasI*) and PAO-JP2 (pKD-*rhlA*) bioreporter strains, respectively. The induction of the bioluminescent strains was monitored using the Luminoskan Ascent Luminometer (Thermo Fisher Scientific, Waltham, MA, USA) set at 37 °C and 490 nm. The measurement was done in white opaque 96-well microtiter plate containing 90 μL of the bacterial culture and 10 μL 3-oxo-C12-HSL for PAO-JP2 (pKD201-*lasI*) or C4-HSL for PAO-JP2 (pKD-*rhlA*). The final concentration range of 3-oxo-C12-HSL was 0.1–1000 nM while that C4-HSL was 0.001–100 µM. As for the control, 90 μL Bacterial cultures and 10 μL LB medium were used. All works were done in triplicates, and the maximum luminescence values were expressed in relative light units (RLU).

### 4.5. Bioluminescence Assay 

The bioluminescence of PAO-JP2 (pKD201-*lasI*) and PAO-JP2 (pKD-*rhlA*) strains were measured at 490 nm and 37 °C for 18 h at 10 min interval with continuously shaken in a white opaque 96-well microtiter plate containing 10 μL of different concentrations of the furanone C-30, 80 μL of the bacterial cultures (at OD_600_ = 0.015) and 10 μL of 3-oxo-C12-HSL (final concentration 100 nM) for PAO-JP2 (pKD201-*lasI*) or 10 μL of C4-HSL (final concentration 10 μM) for PAO-JP2 (pKD-*rhlA*). The final concentrations of furanone C-30 used were 3.125–50 μM. Furanone C-30 was dissolved in DMSO.

### 4.6. Protein Expression and Purification 

The ligand-binding domain of the LasR protein was expressed in *E. coli* BL21-pETM-11 using 0.4 mM isopropyl β-d-1-thiogalactopyranoside (IPTG) at 20 °C overnight in the presence of DMSO, 7.5 μM 3-oxo-C12-HSL or/and 50 μM furanone C-30. The cells’ pellets were obtained at 6000 rpm for 15 min, 4 °C, and resuspended in lysis buffer (50 mM Na_2_H_2_PO_4_ pH 7, 300 mM NaCl, 10 mM imidazole) containing 1 mg mL^−1^ lysozyme and 100 mM PMSF. The resuspended cells were lysed by sonication for 40 s (4-s intervals) with 3-s pulses off at 30% amplitude, and centrifuged at 13,000 rpm for 15 min, 4 °C. The soluble fraction was obtained for purification by nickel-affinity chromatography. 

### 4.7. SDS–PAGE Analysis

The purified protein samples were mixed with sample buffer (Carl Roth) and heated at 95 °C for 5 min before loading onto 12% (*w*/*v*) separating and 4% (*w*/*v*) stacking gels from 37.5:1 acrylamide/bisacrylamide (Bio-Lab). The molecular mass marker used was a 10–180 kDa marker from Bio-Lab. The electrophoresis was run at 100 V for 90 min, and the gels were stained by Instant Blue (Expedeon) for 15 min.

### 4.8. Swarming Motility Assay 

Swarming motility assay was conducted, as described by Banin and co-workers [52], with modifications. The swarming medium was made up of 0.5% (*w*/*v*) casamino acids (Difco), 0.4% (*w*/*v*) glucose, and 62 mM potassium phosphate buffer (pH 7), 7 mM (NH_4_)_2_SO_4_, 2 mM MgSO_4_, 10 µM FeSO_4_, solidified with 0.5% (*w*/*v*) Bacto-agar (Difco) and supplemented with furanone C-30 (50 µM). *P. aeruginosa* PA01 was cultured overnight in M9 medium, and then diluted 1:10 with fresh M9 medium and incubated at 37 °C with agitation until the mid-logarithmic phase (OD_600_ 0.4 to 0.6). Following a brief solidification of the swarm plates, 1 μL *P. aeruginosa* PA01 strain inoculum was spotted on the surface of the agar (at the center). Swarming plates were incubated face up at 37 °C for 16–18 h. Images of the swarming plates were captured with a digital camera and presented appropriately.

### 4.9. Computational Analyses

The 3D conformer of furanone C-30 (PubChem identifier: CID 10131246) in SDF format was downloaded from the PubChem open chemistry database [72,73]. The ligand was docked onto the high-resolution (1.80 Å) crystal structure of the *P. aeruginosa* LasR autoinducer-binding domain complexed with 3-oxo-C12-HSL (PDB identifier: 2UV0) [44] using JAMDA that amalgamates the TrixX docking algorithm [74,75] with the JAMDA scoring function [76]. The protein was prepared by removing all heteroatoms (except those of the structurally relevant water molecules), and the appropriate protonation states and hydrogen coordinates were assigned to the protein via Protoss optimization. The binding site was defined by the native autoinducer, with a site radius of 6.5 Å. Protein–ligand docking was achieved with high precision. Given the absence of atomic coordinates for *P. aeruginosa* RhlR in the PDB, the protein’s autoinducer-binding domain (residues 23–166) was homology-modeled by using the SWISS-MODEL protein structure prediction server [77]. Furanone C-30 was docked onto RhlR in the same manner as described earlier for LasR, except that the binding site was defined by the pocket residues previously predicted by DoGSiteScorer [78].

### 4.10. Statistical Analyses

All statistical analyses were performed using GraphPad Prism version 6.00 for Windows (La Jolla, CA, USA). Results are expressed as means ± SD and *p*-value by Student’s *t*-test. Data points on the graphs are a mean of three different experimental readings to ensure the reproducibility/repeatability of the results.

## Data Availability

The data that support the findings of this study are available upon reasonable request from the authors.

## Supplementary Materials

The following are available online, Figure S1. Induction of bioluminescence.

## Sample Availability

Samples of the compounds are not available from the authors.
