# Supplementary material for: Assessing the Molecular Targets and Mode of Action of Furanone C-30 on Pseudomonas aeruginosa Quorum Sensing
Source: Molecules. 2021 Mar 15;26(6):1620. doi: 10.3390/molecules26061620 (PMC7998126; doi:10.3390/molecules26061620)
Supplement: Supplementary file 1 [file molecules-26-01620-s001.pdf]

Supplementary

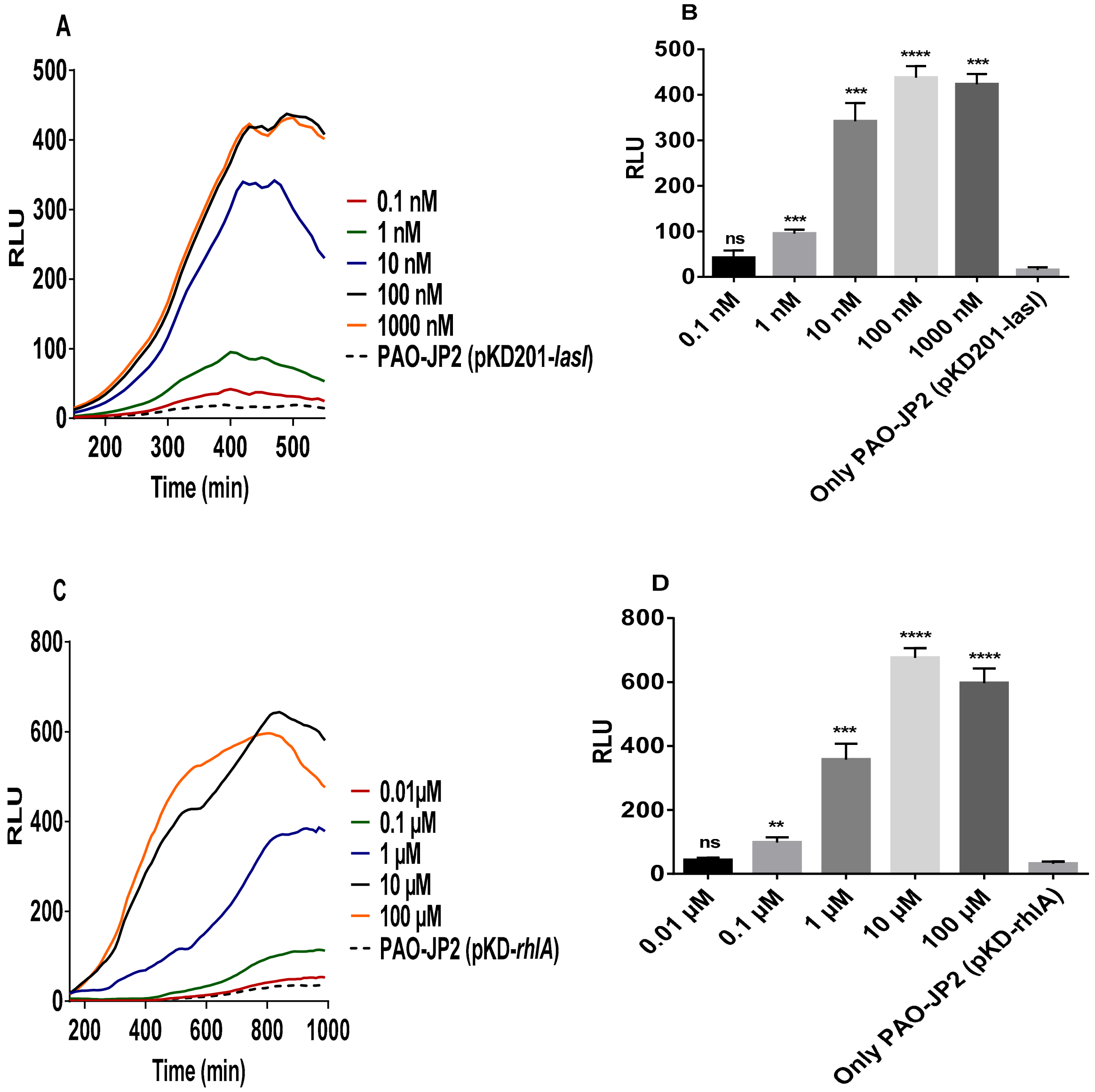

Figure S1. Induction of bioluminescence. **(A)** Bioluminescence emission of PAO-JP2 (pKD201-*lasI*) in the presence of different concentrations of 3-oxo-C12-HSL. **(B)** Bioluminescence induction relative to the control, corresponding to the data in panel A. **(C)** PAO-JP2 (pKD-*rhlA*) luminescence in the presence of different concentrations of C4-HSL. **(D)** Bioluminescence induction relative to the control, corresponding to the data in panel C. All concentrations presented are the final concentrations. Luminescence was expressed as relative light units or RLU. ** $p < 0.01$, *** $p < 0.001$, **** $p < 0.0001$, and *ns* not significant. Values represent mean ± SD, $n = 3$ (three experimental readings).
